# Assessment of the relationship between facial, skeletal, dental and smile asymmetries: a preliminary investigative analysis

**DOI:** 10.1590/2177-6709.30.6.e2524202.oar

**Published:** 2026-02-09

**Authors:** Fernanda de Souza do Nascimento DIOGO, Karoline de Melo MAGALHÃES, Luísa Schubach da Costa BARRETO, Paolla Barboza Araujo de ALMEIDA, Guido Artemio MARAÑÓN-VÁSQUEZ, Luciana Rougemont SQUEFF, Matilde da Cunha Gonçalves NOJIMA

**Affiliations:** 1 Federal University of Rio de Janeiro, School of Dentistry, Department of Pediatric Dentistry and Orthodontics (Rio de Janeiro/RJ, Brazil).; 2 State University of Rio de Janeiro, School of Dentistry, Department of Preventive and Community Dentistry (Rio de Janeiro/RJ, Brazil).

**Keywords:** Facial asymmetry, Orthodontics, Malocclusion, Esthetics, Assimetria facial, Ortodontia, Má oclusão, Estética

## Abstract

**Objective:**

This study aimed to investigate the relationship between asymmetrical faces, smiles, skeletal parameters and dental asymmetries.

**Material and methods:**

A total of 20 patients with normal vertical facial pattern (20≤FMA≤30°) were selected to analyze frontal extraoral photographs with natural smiles and 3D frontal images generated by cone beam computed tomography scans (CBCT scans). The facial symmetry was retrieved by tracing the bipupillary line and perpendicular lines, the distance between the right and left frontolacrimal sutures was subsequently transferred to the same area in the photographs (the intercanthal distance) to calibrate all measures in the study. An assessment of mandibular chin deviation was conducted, and lines were traced to evaluate the dental midline and smile parameters. Through the manipulation of the CBCT scan, the presence of malocclusion and its respective subdivision, as well as the presence of unilateral or bilateral posterior crossbites in each patient, were identified. Data normality was checked using the Shapiro-Wilk test, and univariate linear regressions were performed to assess relationships.

**Results:**

Individuals with Menton point (Me point) deviation greater than 3.5 mm were considered asymmetrical. Subdivision malocclusions significantly correlated with upper midline dental deviation (UMLDD; R^2^= 0.25), with individuals without subdivision malocclusions showing 0.66 mm less deviation (95% CI = -1.24, -0.09; P = 0.026).

**Conclusion:**

UMLDD have a significant correlation with subdivision malocclusion.

## RESUMO

**Objetivos:**

Este estudo teve como objetivo investigar a relação entre assimetrias faciais, parâmetros esqueléticos, assimetrias dentárias e do sorriso.

**Material e Métodos:**

Foram selecionados 20 pacientes com padrão vertical facial normal (20º ≤ FMA ≤ 30º) para análise de fotografias extrabucais frontais com sorriso natural e imagens frontais 3D geradas por tomografia computadorizada de feixe cônico (TCFC). A simetria facial foi avaliada por meio do traçado da linha bipupilar e de linhas perpendiculares. A distância entre as suturas frontolacrimais direita e esquerda foi transferida para a mesma região nas fotografias (distância intercantal), para calibrar todas as medidas. A avaliação da desvio do mento foi realizada, e linhas foram traçadas para análise da linha média dentária e dos parâmetros do sorriso. A partir da manipulação das TCFC, foram identificadas a presença de má oclusão e sua respectiva subdivisão, bem como a ocorrência de mordidas cruzadas posteriores unilaterais ou bilaterais em cada paciente. A normalidade dos dados foi verificada pelo teste de Shapiro-Wilk, e regressões lineares univariadas foram aplicadas para avaliar as relações encontradas.

**Resultados:**

Indivíduos com desvio do ponto Mento (ponto Me) superior a 3,5 mm foram considerados assimétricos. As más oclusões de subdivisão apresentaram correlação significativa com o desvio da linha média dentária superior (DLMDS; R^2^ = 0,25), sendo que indivíduos sem má oclusão de subdivisão apresentaram 0,66 mm a menos de desvio (IC 95%: -1,24 a -0,09; P = 0,026).

**Conclusão:**

O desvio da linha média dentária superior apresenta correlação significativa com a presença de má oclusão de subdivisão.

## INTRODUCTION

The current beauty standards lead society to an incessant pursuit of aesthetic perfection, which is reflected in high expectations regarding harmony of the oral aspect.^1^ The systematic evaluation of facial and dental appearances can be characterized in three steps: macro, mini, and microesthetics. Macroesthetics is represented by facial appearance and parameters such as vertical facial proportions, mandibular asymmetries, retrusive position of the mandible and chin, mandibular prognathism, among others. Miniesthetics encompasses smile structure, including tooth and gingival display, smile arc and width. Microesthetics includes characteristics such as dental proportions and heights, particularly emphasizing the incisal edge’s critical role in defining the aesthetic harmony of the smile line.^2^ ^,^ ^3^

A clinically symmetrical resting face may present slight asymmetry without aesthetic consequences.^4^ However, asymmetries in the nose,^5^ lips,^3^ or chin, ranging from 5 to 10 mm, may be considered unaesthetic.^6^ Asymmetries of 3 mm or more in the smile were perceived by laypersons, and when evaluating smile attractiveness, two-thirds of slightly attractive smiles exhibited asymmetry, while nearly all unattractive smiles were definitively asymmetric.^7^ From a clinical perspective, determining the extent of involvement of craniofacial structures in facial asymmetries is considered important to avoid unexpected misleading of an orthodontic treatment.^8^

Dental asymmetry refers to an imbalance or lack of symmetry in the dental arches, teeth or supporting structures that can affect the way the upper and lower teeth fit together.^2^ The most frequent malocclusion is posterior crossbite and is located between 8% and 23.5% of patients.^9^ Crossbite is the term used to indicate an abnormal buccolingual relationship of the teeth. The most common crossbite is the relationship in which the buccal cusps of some of the posterior upper teeth occlude lingually with the buccal cusps of the lower posterior teeth.^10^ These intraoral dental changes may interfere with bone formation to some extent, subsequently affecting facial formation when not intercepted at an early stage.^11^

There are several conditions associated with facial asymmetry, varying in etiology, severity, clinical course, and treatment.^12^ ^,^ ^13^ Therefore, a thorough understanding of facial asymmetry by the orthodontist is essential to critically analyze the involved characteristics and adequately qualify the magnitude of the disharmony to provide the best possible treatment, taking into consideration the perceptions and expectations of each individual.^5^ ^,^ ^8^ This research hypothesizes that there is a measurable relationship between facial asymmetry, smile characteristics, skeletal parameters, and dental asymmetries, aiming to investigate these connections in a preliminary analysis.

## MATERIAL AND METHODS

This retrospective observational study comprised photographs and three-dimensional imaging examinations from CBCT scans of 20 individuals who began orthodontic treatment at the Orthodontics Postgraduate Clinic, Department of Pediatric Dentistry and Orthodontics, at the School of Dentistry of the Federal University of Rio de Janeiro (UFRJ). This research was approved by the Research Ethics Committee of the Clementino Fraga Filho University Hospital (CEP/HUCFF) at the Federal University of Rio de Janeiro (UFRJ) under approval number 5.751.614, and was conducted and reported in line with the STROBE guidelines^14^ to enhance transparency and reproducibility of observational studies. The sample size of 20 individuals, based on an alpha level of 0.05, utilized the measured difference (mean and standard deviation)^15^ between ANB values obtained through comparison of CBCT scans in a pilot study, providing an estimated power of 80% in this study (with an allowable error of 15%), as described in the referenced article.^7^ ^,^ ^16^

To minimize potential bias, strict inclusion and exclusion criteria were applied, ensuring a homogenous sample in terms of vertical growth patterns (FMA = 20° to 30°) and eliminating individuals with conditions or prior treatments that could interfere with craniofacial development.^16^ The following inclusion criteria were used for sample selection in this study: healthy individuals, regardless of age, race, or sex, in the permanent dentition phase with permanent first molars in occlusion; absence of syndromes; no previous treatment that may have interfered with the normal course of maxillary or mandibular growth and development; normal vertical facial pattern (Frankfort-mandibular plane angle [FMA] values between 20° and 30°)^16^ ^,^ ^17^; initial orthodontic documentation containing frontal extraoral photographs with a posed smile and tomographic files. 

In accordance with the exclusion criteria, individuals with syndromes or a history of craniofacial complex surgery; individuals undergoing orthodontic treatment; individuals in the deciduous or mixed dentition phase; abnormal vertical facial pattern (FMA angle between 20° and 30°); and CBCT scans with artifacts that prevented three-dimensional (3D) analysis were removed from the sample. 

Initially, the individuals’ CBCT scans were standardized by ensuring the patient was in centric relation during the examination and then imported in DICOM format (Digital Imaging and Communications in Medicine) into Dolphin Imaging software (version 11.7 Premium; Dolphin Imaging and Management Solutions, Chatsworth, California, USA), licensed to the Clinic of Orthodontics at UFRJ. Reference planes were defined in the CBCT 3D images.

For head orientation, axial, coronal, and sagittal reference planes were used to make this position similar to the patient’s position in the cephalostat.^17^ The axial plane was determined by the right Orbital (OrR = the lowest point of the lower contour of the right orbit), left Orbital (OrL = the lowest point of the lower contour of the left orbit), and right Porion (PoR = the highest point of the right external auditory meatus). The coronal plane was established by the right Porion (PoR = the highest point of the right external auditory meatus) and left Porion (PoL = the highest point of the left external auditory meatus), perpendicular to the selected axial plane. Finally, the sagittal plane was defined as the point located at the intersection of the frontonasal suture with the internasal suture, perpendicular to the already established axial and coronal planes.^18^

**Figure 1:**
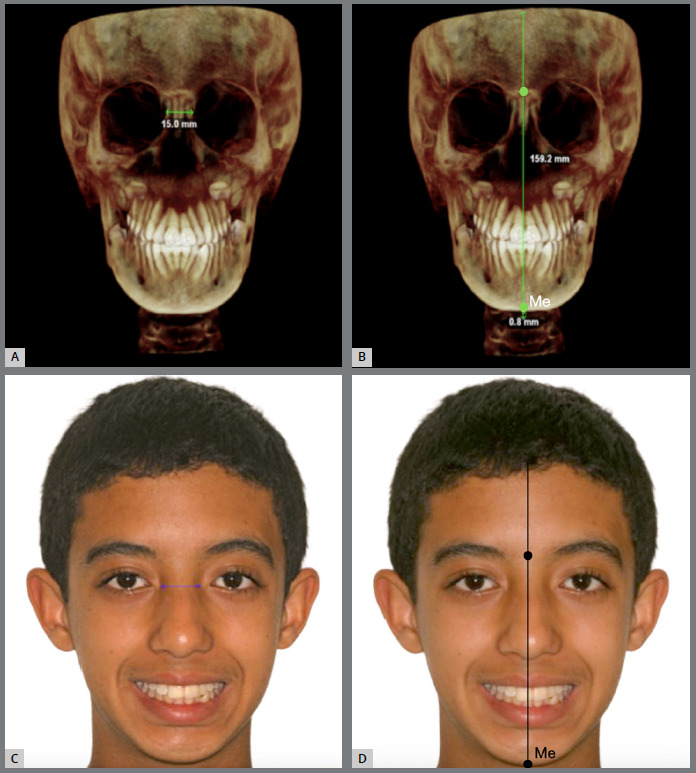
3D images obtained from CBCT scan: **A)** measurement of the distance between the right and left frontolacrimal sutures; **B)** measurement of the distance from the Me point to the VRL. Extraoral photographs: **C)**demonstrating the calibration of the facial photograph, wherein the distance between the frontal lacrimal sutures is transposed to the intercanthal distance on the CBCT scan, with the numerical values being identified accordingly; **D)** evidencing the demarcation of the facial midline. The nasion and the base of the filter (cupid’s bow at the center of the upper lip) served as soft tissue reference points for locating and determining the orientation of the facial midline.

After obtaining the 3D image orientation, measurements were made in the 3D reconstruction image. The distance between the right and left frontolacrimal sutures was measured (Fig. 1A), which was subsequently transferred to the corresponding area, the intercanthal distance in the photographs (Fig. 1C). In addition, a Vertical Reference Line (VRL) described previously,^7^ ^,^ ^16^ ^,^ ^18^ representing the midsagittal plane (a vertical anatomical plane dividing the body into equal right and left halves, often used as a reference for symmetry) was drawn as a black perpendicular line to the line formed between the latero-orbital points passing through the crista galli in the CBCT scan (Fig. 1B). And the distance from the Me point to the VRL was calculated using a horizontal line to analyze the amount of mandibular chin deviation as a quantitative variable, allowing for the analysis of its extent in millimeters.^18^

Subsequently, lateral cephalometric images were generated from the CBCT scans for lateral cephalometric tracing. Individuals with FMA values between 20° and 30° were selected for the study and classified according to sagittal skeletal pattern, through the ANB angle, as: Class I, Class II, and Class III, presenting respectively, 0°≤ANB≤4°; ANB>4°; ANB<0°.^17^

For the classification of dental symmetry (malocclusion symmetry), individuals’ occlusion in the CBCT scans was analyzed. Individuals were assessed according to Angle’s classification and the presence of unilateral or bilateral posterior crossbites as nominal variables to identify the presence or absence of asymmetry. Angle Class II or Class III subdivision malocclusions, as well as unilateral or bilateral posterior crossbites, are asymmetrical malocclusions, and therefore represent dental asymmetries.

Analysis of symmetry in photographic images was performed on initial frontal extraoral photographs of individuals displaying a posed smile. These images were imported into Dolphin Imaging software (version 11.7 Premium; Dolphin Imaging and Management Solutions, Chatsworth, California, USA) in JPG (.jpg) format. Within the software, the images were aligned to ensure that the bipupillary plane was parallel to the ground. Calibration of the photographs was achieved by transferring measurements obtained from the three-dimensional CBCT image (specifically, the distance between the right and left frontolacrimal sutures) to the intercanthal distance, a corresponding soft tissue area in the frontal smile photographs (Fig. 1C). And then, another VRL to divide the right and left sides of the face into photographs (Fig. 1D). 

**Figure 2:**
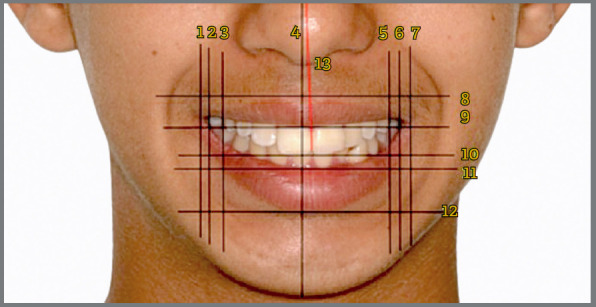
Extraoral facial photograph illustrating the horizontal, vertical, and oblique reference lines utilized in this study for smile analysis. It delineates the inner (1) and outer (2) labial commissures on the right, the distal face of the right upper first premolar (3), the facial midline (4), the distal face of the left upper first premolar (5), the inner (6) and outer (7) labial commissures on the left, the upper border (8) and lower border (9) of the upper lip, the incisal line (10), the upper border (11) and lower border (12) of the lower lip, and the upper dental midline (13).

Subsequently, horizontal lines were drawn parallel to the bipupillary line, including the upper and lower borders of the upper lip, the incisal line, and the upper and lower borders of the lower lip (Fig. 2). Additionally, seven vertical lines were drawn, marking specific anatomical points (Fig. 2) such as the inner and outer labial commissures, the distal faces of the upper first premolars, and the facial midline. An oblique line was also drawn to indicate the upper dental midline, following the methodology proposed by Singh et al.^7^

**Figure 3:**
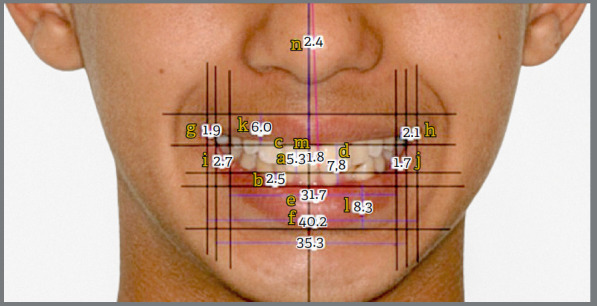
Measurements following previous methodology suggested by Singh et al.^7^, including: visibility of upper (a) and lower (b) incisors, gingival exposure (c), interlabial distance (d), width between upper premolars (e), smile width (f), thickness of right (g) and left (h) labial commissures, right (i) and left (j) mouth corridors, thickness of upper (k) and lower (l) lips, tooth deviation from the upper midline (m), and tooth angulation from the upper midline (n).

After drawing the aforementioned lines, the images were analyzed using Dolphin Imaging software (version 11.7 Premium; Dolphin Imaging and Management Solutions, Chatsworth, California, USA) to obtain various measurements, as per the methodology proposed by Singh et al.^7^ Thirteen points were necessary to obtain seven measurements in this study to assess asymmetry as displayed in Figure 3, and described in Table 1.

**Table 1:** Description of the measures used for asymmetry, according to the previous methodology.^7^

Acronym	Measurement	Definition
LLCT	Left labial commissure thickness	Horizontal distance from the inner labial commissure to the outer labial commissure on the left side
RLCT	Right labial commissure thickness	Horizontal distance from the right inner labial commissure to the right outer labial commissure
LBC	Left buccal corridor	Horizontal distance from the most distal point of the left maxillary first premolar to the inner labial commissure on the left
RBC	Right buccal corridor	Horizontal distance from the most distal point of the right upper first premolar to the inner labial commissure on the right
BCR	Buccal corridor ratio	The ratio of the width between upper premolars divided by the distance between the right and left inner labial commissures
UMLDD	Upper midline dental deviation	Linear discrepancy between the upper dental midline and the facial midline
UMLDA	Upper midline dental angulation	Angle between the upper dental midline and the facial midline

Descriptive statistics summarized sample characteristics. Data normality was checked using the Shapiro-Wilk test to assess data normality due to its sensitivity in small sample sizes, ensuring the appropriateness of subsequent analyses. Univariate linear regressions were applied to explore relationships between variables, as this method effectively identifies associations while maintaining simplicity, which aligns with the investigative nature of the study. Univariate linear regressions evaluated the association between chin deviation, crossbite presence, subdivision of malocclusion and parameters indicating smile asymmetry. Analyses were conducted using Jamovi software version 2.3, with a significance level of 5%.

## RESULTS

The intraclass correlation coefficient (ICC) was computed to evaluate measurement reliability in the three-dimensional images obtained from CBCT. A single investigator (F.S.N.D) measured all variables twice for 20% of the sample, spaced 15 days apart. The ICC exceeded 0.82 for all measured variables, indicating high reliability.

The study sample included 20 individuals: 7 males (35.0%) and 13 females (65.0%). Regarding skeletal malocclusion, 6 participants (30.0%) were classified as Class I, 7 participants (35.0%) as Class II, and 7 (35.0%) as Class III.

Among the 20 individuals studied, the average age was 17.7 (± 7.1) years, with females comprising 65% of the sample. The mean ANB angle, which reflects the sagittal relationship between the maxilla and mandible, was 1.7° (± 3.9°). Regarding vertical pattern, all patients met the study’s inclusion criteria and exhibited an FMA angle ranging from 20 to 30 degrees, indicative of a normal vertical facial pattern. Additionally, the mean Frankfurt mandibular angle was 25.5° (± 4.1°), indicating the overall vertical skeletal pattern of the sample. Individuals with Me point deviation greater than 3.5 mm were considered asymmetrical.^19^ ^,^ ^20^

A significant association was detected between subdivision malocclusions and upper midline dental deviation, as shown in Table 2. Individuals without malocclusion subdivision exhibited a deviation 0.66 mm smaller than those with subdivision (95% CI: -1.24, -0.09; P = 0.026). Malocclusion subdivision accounted for 25% of the variation in upper midline dental deviation in this sample (R^2^= 0.25). No other predictor variables exerted an influence on the remaining smile parameters considered in the present study.

**Table 2:** Univariate linear regression analyses assessing the influence of Me deviation, crossbite, and subdivision malocclusion on smile parameters.

Outcome	Predictor variable	β	95% CI	P value
LLCT	Me deviation	0.05	-0.46, 0.56	0.837
	Crossbite	0.07	-1.42, 1.56	0.920
	Subdivision malocclusion	-0.51	-1.98, 0.96	0.474
RLCT	Me deviation	0.10	-0.55, 0.76	0.746
	Crossbite	1.62	-0.13, 3.37	0.067
	Subdivision malocclusion	-0.11	-2.03, 1.82	0.908
LBC	Me deviation	-0.37	-0.90, 0.17	0.164
	Crossbite	0.42	-1.22, 2.05	0.598
	Subdivision malocclusion	-0.48	-2.11, 1.15	0.541
RBC	Me deviation	-0.11	-0.64, 0.42	0.663
	Crossbite	-0.33	-1.88, 1.21	0.655
	Subdivision malocclusion	-0.28	-1.83, 1.27	0.708
BCR	Me deviation	0.01	-0.01, 0.02	0.400
	Crossbite	0.01	-0.03, 0.05	0.776
	Subdivision malocclusion	0.02	-0.02, 0.06	0.273
UMLDD	Me deviation	-0.11	-0.33, 0.12	0.333
	Crossbite	-0.46	-1.08, 0.17	0.141
	Subdivision malocclusion	-0.66	-1.24, -0.09	0.026*
UMLDA	Me deviation	0.68	-0.26, 1.62	0.147
	Crossbite	-0.94	-3.82, 1.94	0.502
	Subdivision malocclusion	1.42	-1.41, 4.26	0.305

CI = Confidence interval; LLCT = Left labial commissure thickness; RLCT = Right labial commissure thickness; LBC = Left buccal corridor; RBC = Right buccal corridor; BCR = Buccal corridor ratio; UMLDD = Upper midline dental deviation; UMLDA = Upper midline dental angulation.

## DISCUSSION

In this study, the relationship of facial and skeletal asymmetries such as chin deviation was measured on 3D images from CBCT scans, as well as dental and smiles asymmetries were analyzed on extraoral photographs. CBCT imaging offers precise volumetric data of maxillofacial structures, enabling quantification of asymmetries and enhancing clinical assessment accuracy for patients with such conditions,^21^ thus adding significance to our study. The clinical justification for using CBCT scans in this study is based on the orthodontic documentation protocol provided by the University, which includes CBCT as a standardized tool for obtaining precise three-dimensional volumetric data of craniofacial structures.^18^ Additionally, as this is a retrospective study, relying on dental models to assess posterior crossbite could introduce variability influenced by operator fabrication, potentially compromising consistency and accuracy. CBCT scans, therefore, enhance the reliability of the findings, ensuring a more accurate and comprehensive investigation of the relationships between facial, skeletal, and dental asymmetries at a single time point. While the extraoral photographs of individuals displaying a posed smile contribute for a complete analysis of all asymmetries due to layperson perspective of social interaction by frontal communication.^22^

According to Bishara and Burkey’s classification,^12^ facial asymmetries can be indicated as dental, skeletal, muscular, functional, or a combination of these. Dental asymmetries can occur due to the association of local factors, such as dental anomalies, early loss of deciduous teeth, congenitally missing teeth, deleterious oral habits, discrepancies in the midline shift, and occlusal discrepancies. Skeletal asymmetries may involve the maxilla and/or mandible or even skeletal structures on one side of the face. Muscular asymmetries include hemifacial microsomia, Mobius syndrome, cerebral palsy, unilateral hypertrophy of the masseter or temporalis muscle, as well as cases of torticollis involving the sternocleidomastoid muscle, untreated for long periods. Functional asymmetries can be caused by premature contacts that cause lateral displacement of the jaw from the initial contact position to the habitual occlusal position, or the presence of poorly positioned teeth, crossbite, maxillary arch constriction, or anteriorly displaced articular disc.^23^

Methodologically, only individuals exhibiting a normal vertical growth pattern, defined by an FMA angle between 20° and 30°, were included in the present study. With an average FMA angle of 25.5°, facial height exerted no influence on our investigation since there are muscle differences between the various vertical patterns, where individuals with a long face show a more oblique orientation of the muscle fiber bundles^7^ ^,^ ^16^, which could introduce a bias in the present study. Additionally, the balanced distribution among Class I, II, and III skeletal patterns, with an average ANB angle of 1.7°, improved the examination of skeletal, dental, and smile asymmetries across various sagittal skeletal patterns.^18^

Clinical evidence of dental asymmetry, occlusal plane inclination, and midline deviation are frequently found in individuals with facial asymmetry.^22^ ^,^ ^23^ ^,^ ^24^ Midline discrepancies may be isolated or may occur in conjunction with other occlusal asymmetries, particularly molar occlusion asymmetry or Angle’s malocclusion subdivisions^12^ ^,^ ^14^ since those associations can be found in Class II subdivision when it involves distoclusion on one side of the dental arch, and in Class III subdivision when entails mesio-occlusion on one side of the dental arch.^10^

The smile analysis was based on the methodology proposed by Singh et al.,^7^ with posed smile photographs. This selection of effortless posed smile photographs is deemed optimal for recording and assessing smile characteristics due to their reproducibility.^23^

In the study by Bhateja et al.,^21^ which analyzed pre-orthodontic treatment records, some of the most common asymmetries observed among patients not undergoing orthodontic treatment were also considered in our study. These included deviation of the dental line from the facial midline, chin deviation, and molar classification asymmetry, with respective frequencies of 13.4%, 12.1%, and 43.2%.^19^

The present study identified a significant association between subdivision malocclusion and dental deviation from the upper midline (UMLDD), consistent with findings by Sheats et al.,^24^ who observed a statistically significant correlation between molar asymmetry and non-coincidence of midlines in untreated adolescent populations. Additionally, the present study identified malocclusion subdivision accounting for 25% of the variation in UMLDD (R² = 0.25). Individuals without malocclusion subdivisions exhibited 0.66 mm less deviation than those with subdivisions. However, other smile parameters did not show significant relationships with skeletal or dental asymmetry variables. This correlation aligns with Nanda and Margolis’^25^ observations, suggesting that midline discrepancies may occur independently or in conjunction with other occlusal asymmetries,^26^ particularly molar occlusion asymmetry or Angle subdivision malocclusions.^2^ ^,^ ^21^ However, Sanders et al.^27^ found no statistically significant difference in upper dental midline deviation compared between control and Class II subdivision groups.

Complimentary, between 4° and 6° of facial Me deviation, people with horizontal growth patterns were noted significantly for orthodontists and laypeople.^28^ The chin deviation to the left was frequently found in 70-90%,^29^ ^,^ ^30^ but other studies also report prevalent deviation to the right.^31^ Mostly might be due to the differences in study population and evaluation methods. In our study, no significant difference was found in the deviation of the chin (ME point) between the compared groups.

A limitation of the study is the small sample size and the absence of an analysis of the corresponding functional variables to facial asymmetries.^22^ ^,^ ^26^ Given that this was a retrospective preliminary study, it also suggests a larger sample to enhance the inferential power of the statistics.^15^ Nevertheless, the results support a measurable relationship between subdivision malocclusions and UMLDD, emphasizing the need for further exploration of asymmetry in clinical orthodontic practice and measurement bias was minimized by employing validated imaging software and consistent operator techniques. 

Considering the critical role of accurate diagnosis and comprehensive planning for achieving successful outcomes in orthodontic treatment, conducting correlation studies between facial, skeletal, and dental characteristics of malocclusions, including smile asymmetries as explored in this study, is imperative. Given the study’s small and specific sample, the results are not fully generalizable to broader populations. Future studies with larger, more diverse samples and the inclusion of functional variables are necessary to validate and extend these findings. It’s worth noting that this context can profoundly impact individuals’ self-esteem and, consequently, their overall quality of life.

## CONCLUSION

Upper midline dental deviation (UMLDD) demonstrated a significant correlation with the presence of subdivision malocclusions. However, other smile measurements examined in this study did not show significant relationships with dental and skeletal asymmetry variables.
